# High burden of cerebral white matter lesion in 9 Asian cities

**DOI:** 10.1038/s41598-021-90746-x

**Published:** 2021-06-02

**Authors:** Bonnie Yin Ka Lam, Brian Yiu, Encarnita Ampil, Christopher Li-Hsian Chen, Yustiani Dikot, Jacqueline C. Dominguez, Patel Vishal Ganeshbhai, Saima Hilal, Nagaendran Kandiah, SangYun Kim, Jun-Young Lee, Anam Paulus Ong, Vorapun Senanarong, Kam Tat Leung, Huali Wang, Yuan-Han Yang, Tingting Yong, Faheem Arshad, Suvarna Alladi, Samuel Wong, Ho Ko, Alexander Yuk Lun Lau, Vincent Chung Tong Mok

**Affiliations:** 1 Division of Neurology, Department of Medicine and Therapeutics, The Chinese University of Hong Kong, Hong Kong SAR, China; 2 Therese Pei Fong Chow Research Centre for Prevention of Dementia and Margaret Kam Ling Cheung Research Centre for Management of Parkinsonism, Gerald Choa Neuroscience Centre, The Chinese University of Hong Kong, Hong Kong SAR, China; 3 Shenzhen Research Institute, The Chinese University of Hong Kong, Shenzhen, China; 4 Department of Neuroscience and Behavioural Medicine, Faculty of Medicine and Surgery, University of Santo Tomas, Manila, Philippines; 5 Institute for Neurosciences, St. Luke’s Medical Centre, Quezon City, Philippines; 6 Memory Aging and Cognition Center, National University Health System, Singapore, Singapore; 7 Department of Pharmacology, National University of Singapore, Singapore, Singapore; 8 Department of Neurology, Achmad Yani University, Cimahi, Indonesia; 9 Department of Neurology, National Institute of Mental Health and Neuro Sciences, Bengaluru, India; 10 Saw Swee Hock School of Public Health, National University of Singapore and National University Health System, Singapore, Singapore; 11 Department of Neurology, National Neuroscience Institute, Singapore, Singapore; 12 Department of Neurology, Seoul National University College of Medicine, Seoul National University Bundang Hospital, Seoul, South Korea; 13 Department of Psychiatry, SMG-SNU Boramae Medical Centre, Seoul National University College of Medicine, Seoul, South Korea; 14 Department of Neurology, Hasan Sadikin Hospital, Bandung, Indonesia; 15 Department of Medicine At, Mahidol University, Bangkok, Thailand; 16 Dementia Care and Research Centre, Peking University Institute of Mental Health, Beijing, China; 17 Department of Neurology, Kaohsiung Municipal Ta-Tung Hospital and Kaohsiung Medical University Hospital, Kaohsiung Medical University, Kaohsiung, Taiwan; 18 JC School of Public Health and Primary Care, The Chinese University of Hong Kong, Hong Kong SAR, China; 19 Li Ka Shing Institute of Health Sciences, Faculty of Medicine, The Chinese University of Hong Kong, Hong Kong SAR, China

**Keywords:** Cognitive ageing, Diseases of the nervous system

## Abstract

Age-related white matter lesion (WML) is considered a manifestation of sporadic cerebral small vessel disease and an important pathological substrate for dementia. Asia is notable for its large population with a looming dementia epidemic. Yet, the burden of WML and its associated risk factors across different Asian societies are unknown. Subjects from 9 Asian cities (Bangkok, Bandung, Beijing, Bengaluru, Hong Kong, Kaohsiung, Manila, Seoul, and Singapore) were recruited (*n* = 5701) and classified into (i) stroke/transient ischemic attack (TIA), (ii) Alzheimer’s disease (AD)/mild cognitive impairment (MCI), or (iii) control groups. Data on vascular risk factors and cognitive performance were collected. The severity of WML was visually rated on MRI or CT. The prevalence of moderate-to-severe WML was the highest in subjects with stroke/TIA (43.3%). Bandung Indonesia showed the highest prevalence of WML, adjusted for age, sex, education, disease groups, and imaging modality. Hypertension and hyperlipidemia were significant risk factors for WML, and WML was negatively associated with MMSE in all groups. WML is highly prevalent in Asia and is associated with increasing age, hypertension, hyperlipidemia, and worse cognitive performance. Concerted efforts to prevent WML will alleviate the huge dementia burden in the rapidly aging Asian societies.

## Introduction

Asia had the largest number of people suffering from dementia (22.9 million), which was more than twice the numbers in Europe (10.5 million) or the Americas (9.4 million), as recorded in the global impact of dementia in the World Alzheimer Report 2015^1^. This number was estimated to triple to 67 million in 2050—2 to 3 times higher than the estimates for Europe (19 million) or the Americas (30 million)^1^. Devising and implementing preventive strategies against dementia are of paramount importance, particularly in Asia.

Subclinical sporadic cerebral small vessel disease (CSVD) has been increasingly recognized in recent years to be a prevalent and important pathological substrate for cognitive impairment and dementia^2^. Subclinical sporadic CSVD commonly manifests on neuroimaging as white matter lesion (WML), lacunes, microbleeds, enlarged perivascular space, or microinfarcts^3,4^. In patients with stroke, variable severity levels of cognitive impairment are present in more than 80% of patients^5^ and the presence of subclinical CSVD lesion is associated with worse cognitive performance^6–12^. Its presence significantly increases the risk of poststroke dementia^9,13^. Among those who survive stroke without early-onset poststroke dementia, the presence of CSVD can also increase the risk of delayed-onset poststroke dementia^9^.

In patients with Alzheimer’s disease (AD), CSVD is associated with worse cognitive performance and more rapid cognitive decline. Among those with mild cognitive impairment (MCI), it also increases the risk of conversion to AD^14,15^. In addition, some studies have even suggested a causative role of CSVD in AD pathologies^16,17^. Overall, population studies showed that the presence of CSVD increases the risk of incident vascular dementia or AD^18,19^.

Apart from dementia, subclinical CSVD also increases the risk of incident stroke^19^. Other clinical manifestations of CSVD may include depression and other behavioral problems (e.g. apathy), gait and postural instability, and urinary incontinence, which may occur in isolation or in conjunction with dementia/post-stroke syndrome. Overall, CSVD increases the risk of functional decline and mortality^19,20^.

While many risk factors that may be associated with sporadic CSVD, age and hypertension are the most consistent factors^21^. In addition, cerebral WML was found to be highly heritable and recent studies have identified an increasing number of genetic loci associated with CSVD^22^. Given the multi-factorial nature of sporadic CSVD, the burden of CSVD may vary across geographical locations and cultures. The first population-based studies comparing the prevalence of sporadic subclinical CSVD between Asians (Chinese) and Australians suggested that Asians may have a higher prevalence of CSVD than Australians, even after adjusting for vascular risk factors^23^. Although few studies had reported the prevalence of subclinical CSVD in stroke patients in different Asia cities, comparison between studies of different cities was difficult because the studies used different scales for the quantification of CSVD^24–26^. Moreover, very few studies reported the CSVD prevalence in AD in Asia^11^*.* There was limited data on CSVD in some cities such as Indonesia, the Philippines, and Thailand. Finally, no studies had compared the prevalence of subclinical CSVD in stroke and AD subjects in different age groups using a standardized CSVD rating method.

The AWARE (Asian White mAtteR lEsion) study group initiated a joint international effort across multiple Asian centers to estimate the burden of CSVD (using WML as a surrogate marker for CSVD) in Asia. The study group reported the largest community (stroke- and dementia-free subjects) study (*n* = 1797) on the prevalence of subclinical CSVD previously in Asia, which again showed a high prevalence of CSVD in Asia^27^. Apart from community subjects, the AWARE study group also collected data from subjects with stroke/TIA (*n* = 1834) and AD/MCI (*n* = 2070). The objective of the present study was to estimate the burden of cerebral WML in stroke/TIA and AD/MCI across 9 cities in Asia. We also included community control groups from the AWARE to compare against the results of stroke/TIA and AD/MCI groups. We also investigated the risk factors and cognitive impact of WML among all subjects and different clinical groups. The hypotheses were: (i) the overall prevalence of WML in Asia is high but there could be variations in the different Asian cities; (ii) vascular risk factor such as hypertension may be a common contributor to the WML and WML is associated with worse cognitive function across the Asian regions.

## Methods

### Subjects and recruitment procedure

The AWARE study included a total of 5,701 subjects. Subjects consisted of patients with stroke/TIA (*n* = 1834), AD/MCI (*n* = 2070), and controls (*n* = 1797) across 9 Eastern and South-Eastern Asian cities (Bangkok, Bandung, Beijing, Bengaluru, Hong Kong, Kaohsiung, Manila, Seoul, Singapore). Patients with AD dementia or MCI were assessed and recruited in cognitive disorder clinics. Patients were diagnosed with AD dementia according to the NINCDS-ADRDA criteria^28^. Patients were classified as having MCI if there were (i) subjective cognitive complaints; (ii) objective cognitive impairment fulfilled that of local standards in defining cognitive impairment, and (iii) presenting with no or minimal functional impairment in daily functioning. Patients with mixed dementia cases were excluded (n = 197), and hence, none of the patients with AD dementia or MCI had a history of overt stroke. We included both hemorrhagic and ischemic stroke in the stroke group. We defined stroke according to clinical evidence of cerebral injury based on symptoms persisting 24 h or longer, and other etiologies excluded. We defined TIA based on transient neurological deficits (less than 24 h) and the absence of infarcts/hemorrhage on neuroimaging^29^. Based on available data from each cohort, we defined dementia in stroke/TIA patients if they had a clinical dementia rating scale of 1 or above, a mini-mental state examination (MMSE) of 16 or below^30^ or they were diagnosed by respective dementia experts to have vascular dementia according to the NINDS-AIREN criteria^31^. Healthy controls were defined as community-dwelling subjects without history of known cognitive disorder and/or MMSE scored less than education-adjusted cutoff for dementia; without other significant neurological/psychiatric comorbidities, e.g. stroke, multiple sclerosis, psychosis; or severe medical illnesses and were functionally independent^27^.

Ethics approval of each cohort was obtained from the review board of the affiliated university. Ethics approval of the Bangkok cohort was obtained from the Central Institutional Review Board of Mahidol University; the Bandung cohort was obtained from Dr. Hasan Sadikin General Hospital and the Faculty of Medicine, Universitas Padjadjaran Health Research Ethics Committee; the Beijing cohort was obtained from the institutional review board of Peking University Institute of Mental Health (Sixth Hospital); the Bengaluru cohort was obtained from the Nizam’s Institute of Medical Sciences ethics committee; the Hong Kong cohort was obtained from the Joint Chinese University of Hong Kong—New Territories East Cluster Clinical Research Ethics Committee; the Kaohsiung cohort was obtained from the institutional review board of the Kaohsiung Medical University Hospital; the Manila cohort was obtained from the St. Luke’s Medical Center Institutional Ethics Review Committee; the Seoul cohort was obtained from the Institutional Review Board of Seoul National University Bundang Hospital while the Singapore cohort was obtained from the Singapore Eye Research Institute and National Healthcare Group Domain-Specific Review Board.

### Clinical and cognitive measures

Clinical and cognitive measures such as age, sex, education years, and MMSE were collected. Patients were defined as having hypertension if systolic blood pressure ≥ 140 mm Hg and/or diastolic blood pressure ≥ 90 mm Hg, had a history of hypertension, or were using antihypertensive medication. Diabetes mellitus (DM) was defined as a fasting serum glucose level of 7.0 mmol/L or higher, a postprandial serum glucose level of 11.1 mmol/L or higher, or the use of oral hypoglycemic agents/insulin. Hyperlipidemia was defined as a total cholesterol level of 5.2 mmol/L or higher, a low-density lipoprotein cholesterol level of 2.6 mmol/L or higher, a triglyceride level of 1.70 mmol/L or higher, or the use of lipid-lowering drugs^32^.

### Imaging

Subjects with either magnetic resonance imaging (MRI) or computerized tomography (CT) were eligible for this study. For details of the scanner information, please see Supplementary Table S1a,b, and Supplementary Fig. 1. The severity of WMLs was rated on axial MRI FLAIR or CT scan. If both MRI and CT were available, rating was performed on MRI (see Supplementary Table S2).To assess inter-rater agreement between different raters, Hong Kong acted as the central rater, and each center provided 30 raw images to Hong Kong for evaluation of the inter-rater agreement.

### Measurement of WML

The severity of WML for each subject was rated according to the modified Fazekas scale^33^ or the Age-Related White Matter Changes Scale (ARWMC)^34^ based on the MRI axial FLAIR sequence or CT. We used the operationalized global Fazekas rating, which has a score ranging from 0 to 3^35^. We also used the operationalized global ARWMC score, with a score ranging from 0 to 3^34^. We defined the presence of moderate-to-severe WML by a global score of ≥ 2 for both Fazekas and ARWMC scale. The Hong Kong community dataset with both Fazekas and ARWMC global ratings showed that the correlation between the scales was high (*p* < 0.0001; *r* = 0.943). Both visual rating scales of WML showed good agreement between CT and MRI^36^.

### Statistical analysis

Data in continuous variables were first examined by a test of normality. Group comparisons with clinical variables and demographic information were performed using the Kruskal–Wallis test for continuous variables while the chi-square test was used for categorical variables (*p* < 0.05). Post-hoc analyses between cities were conducted. Multiple logistic regression models were used to study the association between vascular risk factors and prevalence of moderate-to-severe WML with adjustment for age, sex and level of education, Asian regions, disease groups, and imaging modality as appropriate. Multiple linear regression models were used to study the association between the prevalence of moderate-to-severe WML and MMSE with adjustment for age, sex, and level of education, Asian regions, and hypertension. In addition, we stratified the subjects into age groups among disease groups (≤ 60, 61–70, 71–80, and ≥ 81 years old) to compare the associations between vascular risk factors and prevalence of moderate-to-severe WML. Pairwise deletion was applied on missing data. Bonferroni correction was used for multiple comparison correction. All analyses were performed using IBM SPSS (IBM SPSS Statistics for Mac OS, Version 24.0).

## Results

**Figure 1:**
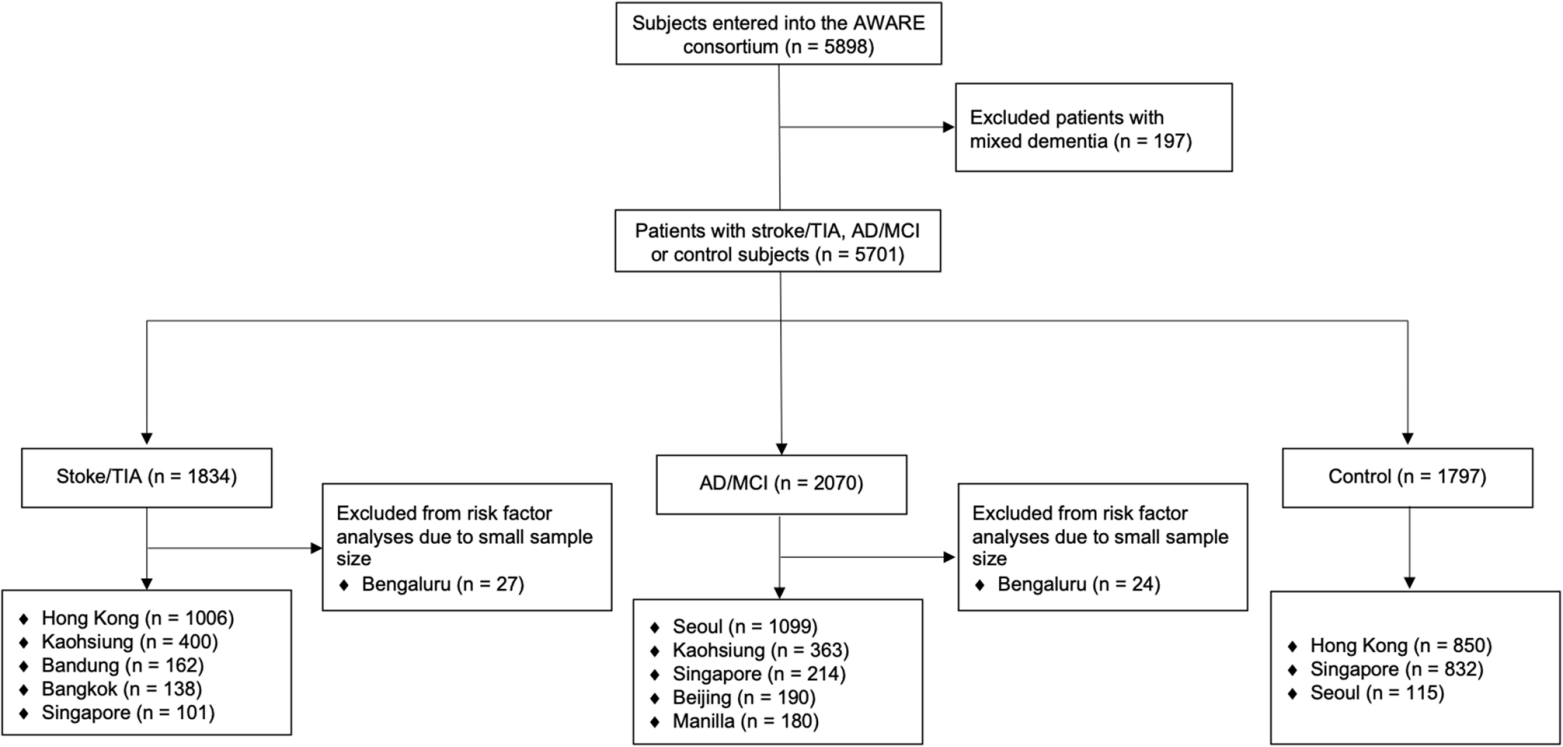
CONSORT diagram of the AWARE study.

The CONSORT diagram shows the study inclusions, as well as the data available from each center (see Fig. 1). Table 1 shows the clinical demographics of the 3 groups: (i) stroke/TIA group; (ii) AD/MCI group, and (iii) control group. The prevalence of moderate-to-severe WML (defined as ≥ 2 on visual rating) in stroke/TIA, AD/MCI and control groups were 43.3%, 38.2% and 36.7% respectively (Table 1).

**Table 1:** Clinical demographics of different disease groups and controls in the AWARE study.

	Stroke/TIA (*n* = 1834)	AD/MCI (*n* = 2070)	Controls (*n* = 1797)	*p*
Age, yr, mean ± SD (min; max)	67.73 ± 12.16 (15; 98)	76.30 ± 8.67 (37; 99)	70.29 ± 6.00 (60; 89)	*p* < 0.001^a,b,c^
Education, yr, mean ± SD (min; max)	6.35 ± 5.02 (0; 30)	8.28 ± 5.57 (0; 22)	7.19 ± 4.79 (0; 22)	*p* < 0.001^a,b,c^
Female; *n* (%)	806 (43.9%)	1319 (63.7%)	1024 (57.0%)	*p* < 0.001^a,b,c^
Hypertension; *n* (%)^i^	1315 (71.9%)	1079 (53.2%)	1220 (67.9%)	*p* < 0.001^a,b,c^
Diabetes mellitus; *n* (%)^ii^	665 (36.4%)	527 (26.1%)	529 (29.5%)	*p* < 0.001^a,b,c^
Hyperlipidemia; n (%)^iii^	1016 (55.8%)	470 (28.2%)	909 (50.6%)	*p* < 0.001^a,b,c^
Ischemic heart disease; *n* (%)^iv^	198 (11.4%)	205 (11.7%)	/	*p* = 0.837
Atrial fibrillation; *n* (%)^v^	258 (14.1%)	42 (2.7%)	/	*p* < 0.001
Smoker (either current or past); *n* (%)^vi^	615 (34.3%)	159 (17.9%)	419 (23.3%)	*p* < 0.001^a,b,c^
MMSE, mean ± SD (min; max)	22.04 ± 6.98 (0; 30)	18.90 ± 6.12 (0; 30)	25.6 ± 3.48 (2; 30)	*p* < 0.001^a,b,c^
WML = 0	499 (27.2%)	269 (13.0%)	258 (14.4%)	*p* < 0.001^a,b,c^
WML = 1	541 (29.5%)	1011 (48.8%)	880 (49.0%)	
WML = 2	416 (22.7%)	495 (23.9%)	533 (29.7%)	
WML = 3	378 (20.6%)	295 (14.3%)	126 (7.0%)	
Prevalence of moderate to severe WML (≥ 2); *n* (%)	794 (43.3%)	790 (38.2%)	659 (36.7%)	*p* < 0.001^a,c^

This table illustrated the clinical demographics of the disease groups and controls in the AWARE study. For post-hoc group comparisons,

^a^AD/MCI versus stroke/TIA.

^b^AD/MCI versus controls.

^c^Stroke/TIA versus controls.

Total number of inclusion cases were ^i^*n* = 5653; ^ii^*n* = 5643; ^iii^*n* = 5286; ^iv^*n* = 3497; ^v^*n* = 3366; ^vi^*n* = 4478.

*MCI* Mild cognitive impairment, *AD* Alzheimer’s disease, *TIA* Transient ischaemic attack, *MMSE* Mini-mental state examination.

Agreement in WML rating standard was first established before data analysis. The Fazekas scale was used as the tool for inter-rater measurement as most countries adopted this scale. The intra-class correlation (ICC) range from fair to excellent in different centers (0.59–0.78), for the ICC results between each center and Hong Kong, please refer to supplementary results.

### Prevalence of WML with age

**Figure 2:**
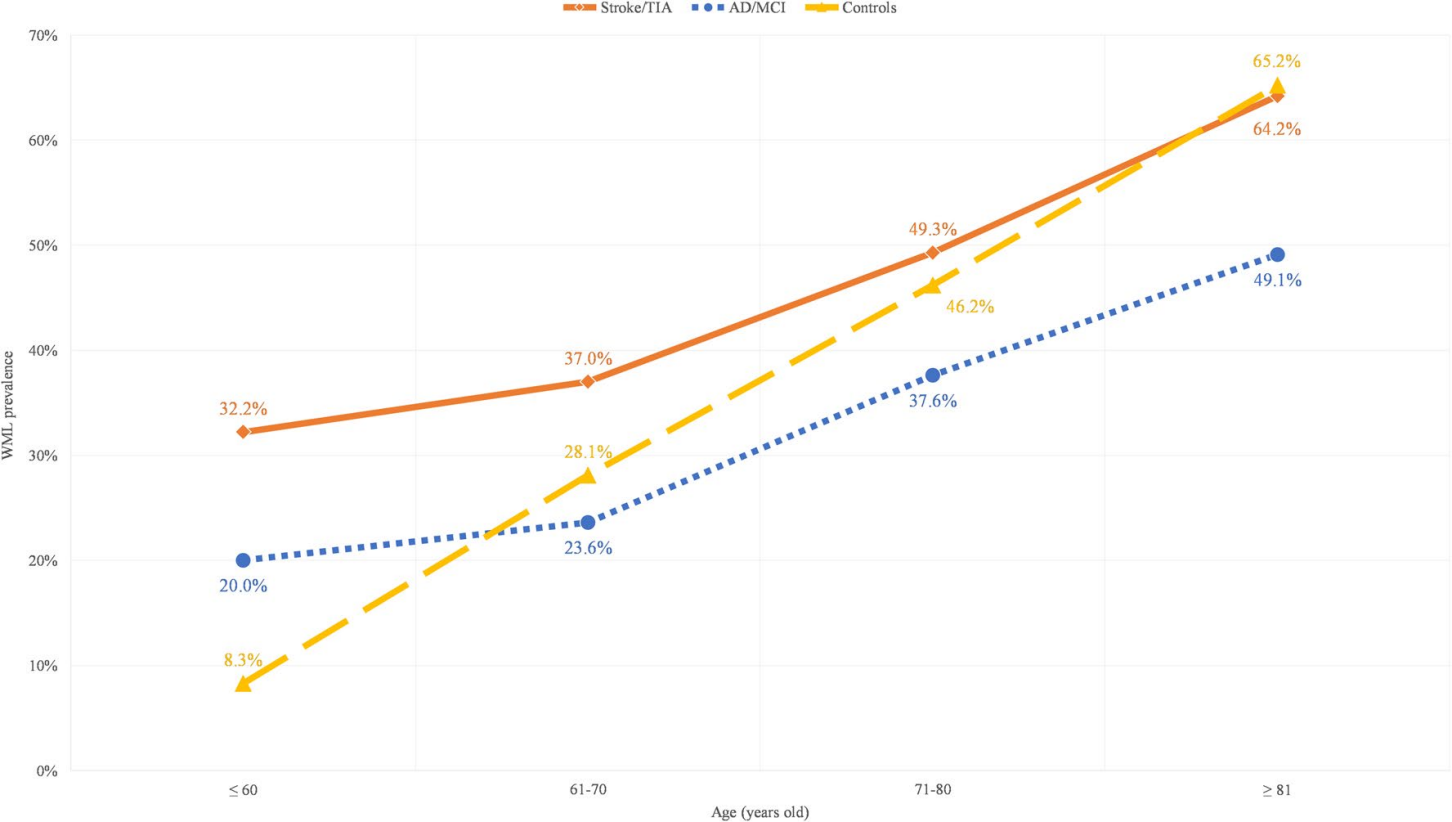
The prevalence of severe white matter lesion in different age groups in the AD/MCI group, stroke/TIA group, and control group.

The prevalence of moderate-to-severe WML in different age groups was examined. Age was divided into four groups and the overall prevalence of moderate-to-severe WML increased with age. The positive trend between age and the prevalence of moderate-to-severe WML was consistent in all groups (Fig. 2). The prevalence of the stroke/TIA group was 32.2% at ≤ 60 years old but increased to 64.2% at ≥ 81 years old. Prevalence of moderate-to-severe WML in the AD/MCI group was 20.0% at ≤ 60 years old and increased at a relatively moderate rate to 49.1% at ≥ 81 years old (Fig. 2). The control group had a relatively lower WML at a younger age (8.3% at ≤ 60 years old) but its prevalence increased proportionally with age and its prevalence reached 65.2%, matching that in the stroke/TIA group at ≥ 81 years old. The age-stratified risk for having moderate-to-severe WML increases with age and was highest in the control group (see Supplementary Table S3).

### Prevalence of WML in specific disease groups and controls

Clinical demographics of various Asian cohorts in specific disease groups and controls were presented in Tables 2, 3 and 4.

**Table 2:** Clinical demographics of the stroke/TIA group.

	Hong Kong (*n* = 1006)	Kaohsiung (*n* = 400)	Bandung (*n* = 162)	Bangkok (*n* = 138)	Singapore (*n* = 101)	p*
Age, year, mean ± SD (min; max)	69.6 ± 11.67 (20; 98)	68.49 ± 12.17 (34; 94)	58.69 ± 11.08 (31; 84)	62.21 ± 13.55 (15; 91)	68.63 ± 7.18 (52; 92)	*p* < 0.001^a,b,f,g,h,i^
Education, year, mean ± SD (min; max)	5.57 ± 4.69 (0; 30)	6.43 ± 5.19 (0; 20)	6.53 ± 4.14 (0; 18)	9.63 ± 5.78 (0; 18)	8.62 ± 4.98 (0; 22)	*p* < 0.001^a,c,d,e,g,h,j^
Female; *n* (%)	445 (44.2%)	157 (39.3%)	90 (55.6%)	55 (39.9%)	53 (52.5%)	*p* = 0.003^i^
Hypertension; *n* (%)	698 (69.4%)	305 (76.3%)	132 (81.5%)	88 (64.2%)	73 (72.3%)	*p* = 0.001^b,e^
Diabetes mellitus; *n* (%)	364 (36.2%)	176 (44.0%)	41 (25.3%)	46 (33.6%)	30 (29.7%)	*p* < 0.001^i^
Hyperlipidemia; *n* (%)	610 (60.6%)	200 (50.0%)	68 (42.0%)	56 (41.2%)	74 (73.3%)	*p* < 0.001^a,b,d,f,h,j^
Ischemic heart disease; *n* (%)	93 (9.2%)	45 (11.3%)	30 (18.5%)	12 (22.2%)	17 (16.8%)	*p* < 0.001^a,b^
Atrial fibrillation; *n* (%)	159 (15.8%)	62 (15.5%)	27 (16.7%)	0 (0.0%)	8 (7.9%)	*p* < 0.001^a,e,f,g^
Smoker (either current or past); *n* (%)	397 (40.0%)	119 (29.8%)	50 (30.9%)	37 (26.8%)	12 (11.9%)	*p* < 0.001^a,c,d,f,h,j^
MMSE, mean ± SD (min; max)	24.05 ± 5.86 (4; 30)	15.24 ± 6.45 (0; 28)	22.93 ± 5.21 (7; 30)	24.35 ± 6.38 (5; 30)	24.56 ± 5.43 (10; 30)	*p* < 0.001^d,g,i,j^
WML = 0	396 (39.4%)	30 (7.5%)	11 (6.8%)	60 (43.5%)	2 (2.0%)	*p* < 0.001^a,b,c,d,e,f,g,h,i^
WML = 1	203 (20.2%)	250 (62.5%)	7 (4.3%)	21 (15.2%)	56 (55.4%)	
WML = 2	203 (20.3%)	86 (21.5%)	29 (17.9%)	57 (41.3%)	34 (33.7%)	
WML = 3	204 (20.3%)	34 (8.5%)	115 (71.0%)	0 (0.0%)	9 (8.9%)	
Prevalence of moderate to severe WML (≥ 2); *n* (%)	407 (40.5%)	120 (30.0%)	144 (88.9%)	57 (41.3%)	43 (42.6%)	*p* < 0.001^b,d,e,h,i^

This table showed the clinical demographics of the stroke/TIA group.

^a^Hong Kong versus Bangkok.

^b^Hong Kong versus Bandung.

^c^Hong Kong versus Singapore.

^d^Hong Kong versus Kaohsiung.

^e^Bangkok versus Bandung.

^f^Bangkok versus Singapore.

^g^Bangkok versus Kaohsiung.

^h^Bandung versus Singapore.

^i^Bandung versus Kaohsiung.

^j^Singapore versus Kaohsiung.

*MMSE* Mini-mental state examination, *WML* White matter lesion.

*Bonferroni correction is applied to the alpha (α = 0.005).

**Table 3:** Clinical demographics of the AD/MCI group.

	Seoul (*n* = 1099)	Kaohsiung (*n* = 363)	Singapore (*n* = 214)	Beijing (*n* = 190)	Manilla (*n* = 180)	p*
Age, year, mean ± SD (min; max)	77.45 ± 7.56 (37; 99)	80.23 ± 8.01 (58; 99)	70.92 ± 9.38 (49; 91)	72.46 ± 7.51 (46; 88)	73.82 ± 9.58 (50; 92)	*p* < 0.001^a,d,e,f,g,h,i,j^
Education, year, mean ± SD (min; max)	7.33 ± 5.33 (0; 20)	6.40 ± 5.21 (0; 20)	7.83 ± 4.83 (0; 22)	12.25 ± 4.68 (0; 21)	13.98 ± 3.40 (0; 20)	*p* < 0.001^a,b,c,d,e,g,h,i,j^
Female; *n* (%)	723 (65.8%)	247 (68.0%)	117 (54.7%)	123 (64.7%)	99 (55.0%)	*p* = 0.001^e,f,i,j^
Hypertension; *n* (%)	491 (44.7%)	167 (47.2%)	120 (59.7%)	63 (36.2%)	113 (63.1%)	*p* < 0.001^a,b,c,i,j^
Diabetes mellitus; *n* (%)	282 (25.7%)	82 (23.7%)	64 (31.8%)	27 (15.5%)	65 (36.1%)	*p* < 0.001^a,b,c,e,i^
Hyperlipidemia; *n* (%)	215 (19.6%)	/	119 (59.2%)	57 (33.1%)	70 (38.9%)	*p* < 0.001^a,c,e,f,h^
Ischemic heart disease; *n* (%)	122 (11.1%)	3 (4.1%)	20 (9.4%)	25 (14.4%)	35 (19.6%)	*p* = 0.002^e,h,i^
Atrial fibrillation; *n* (%)	23 (2.6%)	3 (4.1%)	6 (2.8%)	6 (3.4%)	4 (2.2%)	*p* = 0.900
Smoker (either current or past); *n* (%)	41 (19.0%)	19 (18.1%)	14 (6.5%)	31 (17.9%)	54 (30.0%)	*p* < 0.001^c,f,h,j^
MMSE, mean ± SD (min; max)	18.97 ± 5.58 (0; 30)	15.38 ± 6.38 (0; 28)	20.40 ± 5.58 (3; 30)	20.42 ± 6.46 (1; 30)	21.78 ± 6.08 (5; 30)	*p* < 0.001^a,d,e,f,g,i,j^
WML = 0	182 (16.6%)	57 (15.7%)	15 (7.0%)	2 (1.1%)	12 (6.7%)	*p* < 0.001^a,b,c,d,e,f,g,h,i,j^
WML = 1	570 (51.9%)	94 (25.9%)	75 (35.0%)	118 (62.1%)	134 (74.4%)	
WML = 2	228 (20.7%)	90 (24.8%)	88 (41.1%)	61 (32.1%)	26 (14.4%)	
WML = 3	119 (10.8%)	122 (33.6%)	36 (16.8%)	9 (4.7%)	8 (4.4%)	
Prevalence of moderate to severe WML (≥ 2); *n* (%)	347 (31.6%)	212 (58.4%)	124 (57.9%)	70 (36.8%)	34 (18.9%)	*p* < 0.001^b,c,d,e,f,g,h,i^

This table showed the clinical demographics of the AD/MCI group.

^a^Beijing versus Seoul.

^b^Beijing versus Manilla.

^c^Beijing versus Singapore.

^d^Beijing versus Kaohsiung.

^e^Seoul versus Manilla.

^f^Seoul versus Singapore.

^g^Seoul versus Kaohsiung.

^h^Manilla versus Singapore.

^i^Manilla versus Kaohsiung.

^j^Singapore versus Kaohsiung.

*MMSE* Mini-mental state examination.

*WML* White matter lesion.

*Bonferroni correction is applied to the alpha (α = 0.005).

**Table 4:** Clinical demographics of the control group.

	Hong Kong (*n* = 850)	Singapore (*n* = 832)	Seoul (*n* = 115)	p*
Age, year, mean ± SD (min; max)	71.37 ± 5.09 (64; 89)	69.96 ± 6.46 (60; 88)	64.72 ± 5.31 (60; 79)	*p* < 0.001^a,b,c^
Education, year, mean ± SD (min; max)	7.89 ± 4.91 (0; 22)	6.07 ± 4.52 (0; 22)	10.12 ± 3.37 (6; 18)	*p* < 0.001^a,b,c^
Female; *n* (%)	524 (61.6%)	434 (52.2%)	66 (57.4%)	*p* < 0.001^b^
Hypertension; *n* (%)	519 (61.1%)	669 (80.4%)	32 (27.8%)	*p* < 0.001^a,b,c^
Diabetes mellitus; *n* (%)	202 (23.8%)	311 (37.4%)	16 (13.9%)	*p* < 0.001^b,c^
Hyperlipidemia; *n* (%)	264 (31.1%)	627 (75.4%)	18 (15.7%)	*p* < 0.001^a,b,c^
Smoker (either current or past); *n* (%)	160 (18.8%)	224 (26.9%)	35 (30.4%)	*p* < 0.001^a,b^
MMSE, mean ± SD (min; max)	26.94 ± 2.36 (19; 30)	23.87 ± 3.80 (2; 30)	28.21 ± 1.38 (23; 30)	*p* < 0.001^a,b,c^
WML = 0	170 (20.0%)	25 (3.0%)	63 (54.8%)	*p* < 0.001^a,b,c^
WML = 1	435 (51.2%)	412 (49.5%)	33 (28.7%)	
WML = 2	187 (22.0%)	332 (39.9%)	14 (12.2%)	
WML = 3	58 (6.8%)	63 (7.6%)	5 (4.3%)	
Prevalence of moderate to severe WML (≥ 2); *n* (%)	245 (28.8%)	395 (47.5%)	19 (16.5%)	*p* < 0.001^a,b,c^

This table showed the clinical demographics of the control group.

*MMSE* Mini-mental state examination, *WML* White matter lesion.

^a^Hong Kong versus Seoul.

^b^Hong Kong versus Singapore.

^c^Seoul versus Singapore.

*Bonferroni correction is applied to the alpha (α = 0.0167).

Hong Kong, Kaohsiung, Bandung, Bangkok, Singapore, and Bengaluru contributed data to the stroke/TIA group. When assessing the prevalence of moderate-to-severe WML using the ≥ 2-cutoff, Bandung showed the highest prevalence of moderate-to-severe WML (88.9%) while the Kaohsiung had the lowest prevalence of 30.0% (see Table 2). Data from Bengaluru (*n* = 27) had a relatively small sample size and were excluded from the ANOVA and the subsequent regression analyses. Brief information about the Bengaluru group was specified in the supplementary results.

Seoul, Kaohsiung, Singapore, Beijing, Manila, and Bengaluru contributed data to the AD/MCI group. Among this group, both Kaohsiung and Singapore showed a very high prevalence of WML with gradings 2 or above (58.4% and 57.9% respectively) while Manilla showed the lowest (18.9%) (see Table 3). Data from Bengaluru had a relatively small sample size (*n* = 24) and were excluded from the ANOVA analysis, as well as the subsequent regression analyses. Brief information about the Bengaluru group was included in the supplementary results.

The control group consists of data from Hong Kong, Singapore, and Seoul. Singapore showed the highest prevalence of moderate-to-severe WML (47.5%), followed by Hong Kong (28.8%) while Seoul showed the lowest (16.5%) (see Table 4).

### Risk factors of moderate-to-severe white matter lesion

Multiple logistic regression was performed particularly to ascertain the effects of hypertension, hyperlipidemia, and diabetes mellitus on moderate-to-severe WML on all subjects. Age, sex, level of education, Asian cities, disease groups, and imaging modality were entered as covariates as appropriate. Predictors were only regarded as significant if *p* ≤ 0.0125, corrected for multiple comparisons using Bonferroni correction.

The regression model showed the significant risk factors for moderate-to-severe WML in all subjects were hypertension (*p* < 0.001; OR = 1.81, 95% CI = 1.58–2.08) and hyperlipidemia (*p* = 0.012; OR = 1.19, 95% CI = 1.04–1.36) (Table 5). Hypertension was also a significant predictor for moderate-to-severe WML, after adjustment for age, sex, education and Asian cities and image modality in the stroke/TIA (*p* < 0.001; OR = 1.79, 95% CI = 1.40–2.30), AD/MCI (*p* < 0.001; OR = 1.76, 95% CI = 1.40–2.22) and control groups (*p* < 0.001; OR = 1.97, 95% CI = 1.52–2.55) (Table 5). Strength of association between hypertension and moderate-to-severe WML decreased with increasing age. Similar pattern was observed in all subjects, the stroke/TIA, AD/MCI and control groups (see Supplementary Tables S4–S7).

**Table 5:** Risk factors of moderate-to-severe white matter lesion.

Dependent variables	OR	95% CI		p*
		Lower	Upper	
**All subjects (n = 5215)**				
Age	1.07	1.06	1.08	< 0.001
Female	1.04	0.91	1.18	0.608
Level of education	0.99	0.98	1.00	0.087
HT	1.81	1.58	2.08	< 0.001
DM	1.10	0.96	1.26	0.163
HLD	1.19	1.04	1.36	0.012
Hong Kong (reference)	/	/	/	< 0.001
Beijing	0.95	0.60	1.51	0.816
Bangkok	2.08	1.38	3.14	0.001
Bandung	45.41	25.72	80.19	< 0.001
Seoul Korea	0.48	0.34	0.68	< 0.001
Manilla	0.26	0.15	0.44	< 0.001
Singapore	1.81	1.49	2.20	< 0.001
Kaohsiung	0.48	0.35	0.66	< 0.001
Control (reference)	/	/	/	< 0.001
AD/MCI	1.54	1.14	2.08	0.005
Stroke/TIA	1.90	1.49	2.43	< 0.001
CT (reference)	/	/	/	< 0.001
MRI 1.5 T	1.37	1.00	1.86	0.047
MRI 3 T	1.75	1.33	2.32	< 0.001
**Stroke/TIA (n = 1786)**				
Age	1.07	1.06	1.08	< 0.001
Female	1.26	1.01	1.57	0.040
Level of education	0.99	0.97	1.02	0.493
HT	1.79	1.40	2.30	< 0.001
DM	1.08	0.87	1.35	0.482
HLD	1.21	0.97	1.51	0.092
Hong Kong (reference)	/	/	/	0.000
Bangkok	1.76	1.15	2.70	0.010
Bandung	44.01	24.28	79.77	< 0.001
Singapore	0.72	0.44	1.19	0.200
Kaohsiung	0.37	0.26	0.52	< 0.001
CT (reference)	/	/	/	< 0.001
MRI 1.5 T	1.23	0.89	1.70	0.216
MRI 3 T	2.19	1.59	3.00	< 0.001
**AD/MCI (n = 1633)**				
Age	1.06	1.05	1.08	< 0.001
Female	0.81	0.63	1.04	0.095
Level of education	1.01	0.98	1.03	0.643
HT	1.76	1.40	2.22	< 0.001
DM	1.07	0.83	1.38	0.602
HLD	1.12	0.86	1.45	0.404
Beijing (reference)	/	/	/	< 0.001
Seoul Korea	0.55	0.38	0.81	0.002
Manilla	0.29	0.17	0.48	< 0.001
Singapore	2.71	1.69	4.35	< 0.001
CT (reference)	/	/	/	0.993
MRI 1.5 T	/	/	/	0.999
MRI 3 T	/	/	/	0.999
**Controls (n = 1796)**				
Age	1.10	1.08	1.12	< 0.001
Female	0.94	0.75	1.18	0.591
Level of education	0.98	0.96	1.01	0.184
HT	1.97	1.52	2.55	< 0.001
DM	1.15	0.91	1.45	0.233
HLD	1.12	0.88	1.43	0.356
Hong Kong (reference)	/	/	/	< 0.001
Seoul Korea	1.13	0.65	1.95	0.671
Singapore	2.18	1.71	2.79	< 0.001

This table shows the vascular risk factors contributing to moderate-to-severe white matter lesion. Age, sex, level of education, Asian cities, disease groups, and image modality are entered as covariates in each regression model as appropriate.

*DM* Diabetes mellitus, *HLD* Hyperlipidemia, *HT* Hypertension.

*Bonferroni correction is applied to the alpha (*α* = 0.0125).

The association between hyperlipidemia and moderate-to-severe WML was significant in all subjects (*p* = 0.012; OR = 1.19, 95% CI = 1.04–1.36), as well as in those in the 61–70 age range (*p* = 0.049; OR = 1.28, 95% CI = 1.00–1.64), (Table 5 and Supplementary Table S4). However, the association in the 61–70 age group did not survive Bonferroni correction for multiple comparison. There was no significant association between diabetes mellitus and moderate-to-severe WML in all disease groups.

### The risk of moderate-to-severe WML in different Asian regions

To examine the variations of WML in different regions and account for sampling bias in each region, the regression was performed with adjustment for age, sex and level of education, Asian regions, disease groups, and imaging modality (see Table 5). Using Hong Kong as a reference center, Bandung showed a higher risk of having moderate-to-severe WML (*p* < 0.001; OR = 45.41, 95% CI = 25.72–80.19) while Manila showed a lower risk (*p* < 0.001; OR = 0.26, 95% CI = 0.15–0.44) when all groups were combined.

When focusing on the stroke/TIA group, Bandung showed a higher risk of having moderate-to-severe WML (*p* < 0.001; OR = 44.01, 95% *CI* = 24.28–79.77) while Kaohsiung showed a lower risk (*p* = 0.001; OR = 0.37, 95% *CI* = 0.26–0.52) compared to Hong Kong. Using Beijing as a reference center, Singapore showed a higher risk of having moderate-to-severe WML (*p* < 0.001; OR = 2.71, 95% *CI* = 1.69–4.35) while Manila showed a lower risk of WML (*p* < 0.001; OR = 0.29, 95% *CI* = 0.17–0.48) in the AD/MCI group. Finally, Singapore showed a higher risk of having moderate-to-severe WML compared to Hong Kong and Seoul (*p* < 0.001; OR = 2.18, 95% CI = 1.71–2.79).

### Moderate-to-severe WML and cognition

Multiplelinear regression analyses showed that the presence of moderate-to-severe WML was significantly associated with lower MMSE scores among all subjects and among each disease group (see Table 6). Overall, the MMSE was 20.6 ± 6.2 in those with moderate-to-severe WML (*n* = 2529) compared to 22.5 ± 6.5 in those none-or-mild WML (*n* = 3621). Further, subgroup analysis showed that MMSE was approximately 2 points lower in those with moderate-to-severe WML compared to those without. The MMSE scores in the moderate-to-severe WML and none-or-mild WML were 20.4 ± 6.9 and 22.6 ± 7.0 respectively in the stroke/TIA group; 17.5 ± 6.2 and 19.7 ± 5.9 respectively in the AD/MCI group; and 24.5 ± 3.9 and 26.3 ± 3.0 respectively in the control group. Subjects with stroke/TIA and dementia had a higher prevalence of moderate-to-severe WML than those with stroke/TIA but without dementia (51.7% vs 40.7%). Subjects with AD dementia also had a higher prevalence of moderate-to-severe WML than those with MCI (41.2% vs 25.3%).

**Table 6:** Association between MMSE and moderate-to-severe white matter lesion.

n	β	95% CI for B		p*
		Lower	Upper	
**All subjects**				
5519	− 0.052	− 0.945	− 0.415	< 0.001
**Stroke/TIA**				
1765	− 0.056	− 1.316	− 0.258	0.004
**AD/MCI**				
1958	− 0.079	− 1.478	− 0.517	< 0.001
**Controls**				
1796	− 0.064	− 0.735	− 0.191	0.001

This table shows the association between moderate-to-severe white matter lesion contributing to cognition. Age, sex, level of education, Asian cities and hypertension are entered as covariates in each regression model.

*MMSE* Mini-mental state examination.

*Bonferroni correction is applied to the alpha (*α* = 0.0125).

## Discussion

This is the first multi-center study to formally assess the prevalence of moderate-to-severe WML in stroke/TIA and AD/MCI, in different age groups, and from 9 Asian cohorts. The prevalence of moderate-to-severe WML was higher in stroke/TIA (43.3%) than that in AD/MCI (38.2%), ${\text{X}}_{(1)}^{2}$ = 10.6, *p* = 0.001. Moderate-to-severe WML was associated with hypertension, hyperlipidemia, and a lower MMSE score.

In this study, we primarily reported the prevalence of moderate-to-severe WML, rather than any presence of WML. Previous studies that included any presence of WML (i.e. including those with mild WML as well) reported a much higher prevalence of WML, reaching 81.4% in the community^37^. We recorded only moderate-to-severe WML because previous studies showed that only those with moderate-to-severe WML as defined by a grade 2 or above in the global rating of Fazekas or ARMCW scale were associated with increased risk of incident cognitive decline. Although mild or focal/punctate WML may still represent early or minor SVD, previous studies showed that it had no or minimal clinical relevance, while longitudinal studies revealed no progression of mild WML over time^35^. Whereas, early confluent to confluent WML will likely progress in size and is clinically relevant^15,18,20,35^. In cohorts where the prevalence of mild WML is high and that of moderate-to-severe WML is low, reporting any presence of WML may overestimate the severity of CSVD.

### WML in stroke/TIA

Despite the mean age of the stroke/TIA group was the youngest (67.7 ± 12.2 years old) when compared with the AD/MCI group (76.3 ± 8.7 years old) and controls (70.3 ± 6.0 years old), the stroke/TIA group had a higher prevalence of moderate-to-severe WML to that of AD/MCI group as a whole and at each age group, with a prevalence of 32.2% for age ≤ 60 that increased to 64.2% for subjects older than 80 years (Fig. 2). The high prevalence of moderate-to-severe WML in stroke/TIA was most probably explained to a large extent by the fact that both stroke/TIA and WML shared a strong association with cardiovascular risk factors, in particular hypertension^38^. The most alarming finding was observed in Bandung, Indonesia where the prevalence of moderate-to-severe WML is highest among all cities. In particular, hypertension (81.5%) in this group was high despite the relatively young mean age (59 years old). In addition, the Indonesian cohort provided only CT imaging and hence we might have even underestimated the prevalence of moderate-to-severe WML, as CT is less sensitive in the detection of WML relative to MRI. This is indeed the first study investigating the prevalence of WML in Indonesia, which showed a very high prevalence of WML in this region, alongside a high prevalence of hypertension.

### WML in AD/MCI

The prevalence of moderate-to-severe WML in the AD/MCI group was lower than that in stroke/TIA across all age distributions by about 12–15%. Still, the prevalence ranged from 20% for age ≤ 60 to almost 50% for subjects older than 80 years old. The prevalence of HT and other cardiovascular risk factors were lower in AD/MCI than that in the stroke/TIA group, which might at least partially explain the lower prevalence of WML in AD/MCI than in the stroke/TIA group. Another interesting observation was seen in Manila, despite having a large proportion of patients having hypertension (63.1%) and being a current or a past smoker (30%), only 18.9% of the patients in Manila had the lowest prevalence of moderate-to-severe WML. Note that all subjects in the Manila cohort had MRI, and hence, under-reporting of WML was not likely. Whether Filipinos are less susceptible to the development of WML in association with hypertension requires further investigation^39^.

### WML in the control group

Our previous report on the prevalence of CSVD in Asian communities did not report specifically the prevalence of moderate-to-severe WML according to different age groups^27^. In the current study, the prevalence of moderate-to-severe WML rose from 28.1% at 61–70 years old to 65.2% at ≥ 81 years old. Of note, this prevalence of WML is even slightly higher than the prevalence rates of amyloid positivity reported previously among subjects with normal cognition, which vary from around 20% at 70 years old to slightly above 40% at 90 years old^40^. Among the three cities with community subjects, Singapore recorded the highest prevalence of moderate-to-severe WML despite a mean age of only 69.96 years old, which could again be related to the fact that the Singapore group had the highest prevalence of hypertension (80.4%) and hyperlipidemia (75.4%) relative to other cities.

Previous studies on the prevalence of WML have been conducted mainly among Europe and North America. Existing studies showed that WML is almost endemic among the elderly. In the general population, the prevalence of WML was 39% in the Helsinki Aging Brain Study. In particular, WML prevalence was detected in 21% in those < 75 years old and 65% in those ≥ 75 years old^41^. In the oldest-old (80–90 years), either subcortical and periventricular WML was detected in ≥ 95% of the subjects in the Rotterdam Scan Study^42^. When assessing WML longitudinally within the same cohort, the Austrian Stroke Prevention Study showed that the median increase of WML volume to reach early confluent to confluent WML was 36% at the 3-year follow-up and 58% at the 6-year follow-up^43^. The incidence and prevalence of WML vary depending on numerous factors including the age, sex of the subject, whether the subject is healthy or has experienced stroke or dementia, the quality of image acquired, as well as the WML rating method. Thus, direct comparisons or interpretation of the results remain challenging.

### Association between WML, vascular risk factors and global cognition

We found that the strength of association between hypertension and moderate-to-severe WML decreased with increasing age, which suggested that the effects of blood pressure-lowering therapy may be more effective in the younger age group. While among older patients, factors other than high blood pressure (e.g. impaired autoregulation) may have a greater contribution to WML^44^. Note further that the strength of association between hypertension and WML in the AD/MCI group (*p* < 0.001; OR = 1.76, 95% CI = 1.40–2.22) was similar to that of in the stroke/TIA (*p* < 0.001; OR = 1.79, 95% CI = 1.40–2.30) or control groups (*p* < 0.001; OR = 1.97, 95% CI = 1.52–2.55), suggesting that moderate-to-severe WML observed in subjects with AD/MCI may have a significant vascular component.

Previous studies showed conflicting results concerning the association between hyperlipidemia and WML^45,46^. The present study with a large sample size of approximately 6000 subjects did show a small yet significant association between hyperlipidemia and moderate-to-severe WML. The strength of association was less than that for hypertension. Similar to most previous studies, we could not demonstrate an association between DM and WML. Note, however, that other studies showed that DM was related to measures of lacunes, rather than to WML^47–49^.

Above all, we have shown that moderate-to-severe WML was associated with worse cognitive performance in stroke/TIA, AD/MCI and controls. MMSE of those with moderate-to-severe WML was approximately 2 points lower compared to those without moderate-to-severe WML among all subjects and among each of the clinical groups.

### Strengths and limitations

The strength of this study includes the large sample size, the inclusion of the AD/MCI and stroke/TIA groups that are known with a high prevalence of moderate-to-severe WML, as well as a large control group for comparison. Further, this is the first joint international effort with standardized measurement of the WML burden across multiple clinical cohorts in different Asian cities. In addition, analyses in this study were also adjusted with confounders such as age, sex, education, vascular risk factors, disease groups, and different Asian cities.

However, there are certain limitations in this study. First, we acknowledge that certain biases were introduced in the study. Similar to any multi-center study, the pooled sample may contain a more heterogeneous dataset across centers, as well as a potential source of sampling bias from non-random recruitment. However, we have set the study-specific inclusion and exclusion criteria and used the same neuropsychological screening test (MMSE). Survival bias may be introduced as the patients enrolled in the study could have been biased to the younger group who had better cognitive function, and milder chronic brain changes. This could lead to an underestimation of the true magnitude of the prevalence of WML in each city, as well as the association between WML, vascular risk factors, and cognition.

Second, we have only used a brief screening test to measure the overall cognitive function. MMSE is not sensitive in detecting executive dysfunction or slow processing speed that are predominantly affected by CSVD. Further, the MMSE was administered in various languages and cultures specific to the local population and there may be characteristics differences in each version^50^. Third, we acknowledge that Fazekas and ARWMC are two different visual rating scales for WML. Although the scales are different, a cut-off at ≥ 2 in both scales was selected as they both indicated a certain severity of WML that is progressive, and thus, malignant^43^, and lead to devastating outcomes such as global functional decline or delayed-onset dementia^18,20,51^. The Hong Kong community dataset with both Fazekas and ARWMC global ratings showed that the correlation between the scales was high (*p* < 0.0001; *r* = 0.943).

Forth, WML can be associated with inflammation, cerebral amyloid angiopathy, or neurodegenerative disease secondary to Wallerian degeneration, which we were unable to differentiate in this study. Fifth, the use of antihypertensive medications may delay the progression of WML^52^. Most subjects with hypertension included in this study received treatment. However, there is no sufficient data collected to provide further information on how well the hypertension was controlled or types of medication used. Similarly, the management of hypertension may vary among regions and may confer different risks. Future studies should take more detailed information about types of medication used and whether hypertension was controlled, in addition to objective measures of systolic and diastolic blood pressure. Sixth, although we had included data from Bengaluru India to investigate the prevalence of WML and vascular risk factors, the small sample size prevented us from entering the data in regression analyses. Finally, other CSVD imaging markers such as lacunes, cerebral microbleeds, enlarged perivascular space, and microinfarcts were not assessed and may have impact on the overall clinical manifestation.

### Implications

Our findings that there were high burden of CSVD/WMLs in stroke/TIA, AD/MCI, community controls, and specific cities (e.g. Indonesian, Singaporean), and that it was associated with hypertension, hyperlipidemia, and poor global cognition. These findings have huge implications in the management, research and public health strategies for preventing dementia in Asia. Strategies that can improve management of high blood pressure and lipids at both population and individual levels cannot only prevent stroke but also potentially prevent or delay the development and progression of subclinical CSVD/WML. Recent clinical trials of large sample sizes and long durations did show positive effects of aggressive blood pressure lowering and use of statins upon reducing the progression of WML, along with possible benefits upon cognition^53–57^. Strategies targeting CSVD should also incorporate other measures relevant to dementia prevention (e.g. reduce air pollution, limit alcohol, avoid smoking, provide primary and secondary education, introduce a healthy diet, reduce obesity and diabetes, increase physical exercise, and improve sleep quality, etc.) to achieve the maximal effect in reducing dementia burden. Management of hypertension, in particular, should start in mid-life as the evolution of WML from no to severe WML may take more than a decade. Such comprehensive strategies if can be implemented aggressively and efficiently in cities with a high burden of vascular risk factors and CSVD (e.g. Indonesia), the effect size in preventing dementia cases in these cities may be more pronounced. In addition to primary preventive strategies, we propose that more clinical trials should target individuals who already harbor moderate-to-severe CSVD/WML at different clinical contexts, e.g. poststroke/TIA with cognitive impairment (without dementia), MCI (prodromal AD), or even in “preclinical CSVD” dementia- and stroke-free individuals, as they are at high risk of further cognitive decline or dementia*.* Preferably, such trials should include Asians so that findings from these trials can be generalized to Asians as well.

## Supplementary information

Supplementary Information.
