# Supplementary material for: High burden of cerebral white matter lesion in 9 Asian cities
Source: Sci Rep. 2021 Jun 2;11:11587. doi: 10.1038/s41598-021-90746-x (PMC8172636; doi:10.1038/s41598-021-90746-x)
Supplement: Supplementary file 1 — Supplementary Information. [file 41598_2021_90746_MOESM1_ESM.docx]

**Supplementary Table S1a**

|  | Hong Kong | | Beijing | Bangkok | Bengaluru | Seoul | | Manila | Singapore | | | Kaohsiung | |
| --- | --- | --- | --- | --- | --- | --- | --- | --- | --- | --- | --- | --- | --- |
|  | Stroke/TIA | Control | AD/MCI | Stroke/TIA | AD/MCI | AD/MCI | AD/MCI | AD/MCI | Stroke/TIA | AD/MCI | Control | Stroke/TIA | AD/MCI |
| Vendor | Philips | Philips | Siemens | Philips | Siemens | Philips | Philips | General Electrics | Siemens | Siemens | Siemens | General Electrics | General Electrics |
| Model | Achieva | Achieva | Magnetom Trio Tim | Intera | Magnetom Skyra | Intera | Achieva | Signa Pioneers | Magnetom Trio Tim | Magnetom Trio Tim | Magnetom Trio Tim | Signa HDxt | Signa HDxt |
| Magnetic field strength | 3T | 3T | 3T | 1.5T | 1.5T & 3T | 1.5T | 3T | 3T | 3T | 3T | 3T | 1.5T | 1.5T |

**Supplementary Table S1b**

|  |  |  |  |  |  |  |  |
| --- | --- | --- | --- | --- | --- | --- | --- |
|  | Hong Kong | Bangkok | Bandung | Kaohsiung | Singapore | | Bengaluru |
|  | Stroke/TIA | Stroke/TIA | Stroke/TIA | AD/MCI | Stroke/TIA | AD/MCI | Stroke/TIA |
| **Vendor** | GE Healthcare Systems | GE Healthcare Systems | Hitachi | Toshiba | Philips | Philips | Siemens |
| **Model** | LightSpeed VCT | LightSpeed 16 | Scenaria | Aquilion One Vision | Brilliance | Brilliance | Somatom go.Top |
| **No of detector-row** | 512 | 256 | 128 | 320 | 256 | 256 | 128 |

Supplementary Table 1a & b showed the details of MRI and CT scanner information used in each Asian region.

**Supplementary Figure 1**


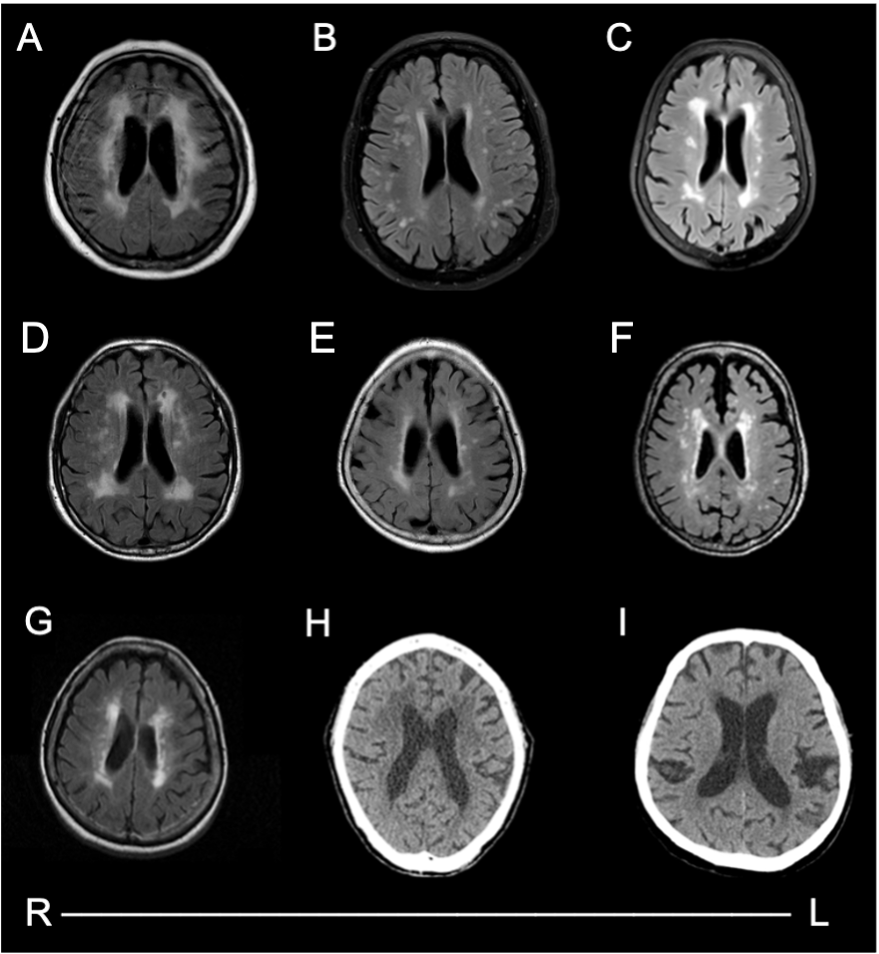


Supplementary Figure 1 - Representative images acquired from MRI and CT in different regions.

Images A-F showed images of MRI from (A) Hong Kong, (B) Beijing, (C) Bengaluru, (D) Seoul (1.5T), (E) Seoul (3T), (F) Singapore, and (G) Kaohsiung. Images H & I showed images of CT from Hong Kong and Singapore, respectively. Selection of image was based on the availability and consent given from the respective site.

| **Supplementary Table S2** | | |
| --- | --- | --- |
|  | MRI (n) | CT (n) |
| Hong Kong | 1367 | 489 |
| Seoul | 1214 | 0 |
| Singapore | 1142 | 5 |
| Kaohsiung | 426 | 337 |
| Beijing | 190 | 0 |
| Manilla | 180 | 0 |
| Bangkok | 55 | 83 |
| Bengaluru | 50 | 1 |
| Bandung | 0 | 162 |

Supplementary Table S2 shows the number of MRI and CT scans performed in each site.

**Supplementary Results**

**Agreement on WML rating**

The intra-class correlation, p-value, and 95% confidence interval (CI) for WML between Hong Kong and the other centers were as follow:

The correlation was fair in Seoul, Korea (0.59, *p* = 0.001, 0.24 – 0.78), good in Bangkok, Thailand (0.61, *p* = 0.003, 0.20 – 0.81); Kaohsiung, Taiwan (0.66, *p* < 0.001, 0.40 – 0.82); Bengaluru, Indian (0.68, *p* < 0.001, 0.43 – 0.84); Bandung, Indonesia (0.71, *p* = 0.008, 0.15 – 0.89); and excellent in Manila, the Philippines (0.75, *p* < 0.001, 0.53 – 0.87); Singapore (0.76, *p* < 0.001, 0.54 – 0.88); and Beijing, China (0.78, *p* < 0.001, 0.58 – 0.89).^1^

**Additional information for Table 2a**

Brief information about the Bengaluru group was as follow: mean age was 65.85 ± 11.70 years old, mean years of education were 8.59 ± 5.98; 22.2 *%* were female, and the prevalence of WML was 0.0 *%* with grade 0, 14.8 *%* with grade 1, 25.9 *%* with grade 2, and 59.3 *%* with grade 3.

**Additional information for Table 2b**

Brief information about the Bengaluru group was as follow: mean age was 61.29 ± 6.95 years old, mean years of education were 8.11 ± 5.59; 41.7 *%* were female, and the prevalence of moderate-to-severe WML was 4.2 *%* with grade 0, 83.3 *%* with grade 1, 8.3 *%* with grade 2, and 4.2 *%* with grade 3.

**Supplementary Table S3**

Age-stratified risk on the prevalence of WML in different disease groups

|  | Aged <=60 | Aged 61-70 | Aged 71-80 | Aged >=81 | Wald X^2^ test |
| --- | --- | --- | --- | --- | --- |
| Stroke/TIA | Reference †,‡ | 1.24 (0.96 - 1.60) †,‡ | 2.05 (1.59 - 2.64) ‡ | 3.78 (2.77 - 5.16) | X^2^_(3)_ = 86.35; p <0.001 |
| AD/MCI | Reference †,‡ | 1.24 (0.73 - 2.08) †,‡ | 2.41 (1.50 - 3.88) ‡ | 3.86 (2.39 - 6.25) | X^2^_(3)_ = 78.67; p <0.001 |
| Control | Reference *,†,‡ | 4.29 (1.70 - 10.83) †,‡ | 9.43 (3.73 - 23.85) ‡ | 20.59 (7.62 - 55.67) | X^2^_(3)_ = 106.16; p <0.001 |

**Note:** * denotes *p* <.05 vs 61-70 age; † denotes *p* <.05 vs 71-80 age group; ‡ denotes *p* <.05 vs >=81 age group

**Supplementary Table S4**

| **All subjects (n=5215)** | | | | |  |
| --- | --- | --- | --- | --- | --- |
| Dependent variables | OR | 95% CI | | **p* |  |
|  |  | Lower | Upper |  |  |
| Age | 1.07 | 1.06 | 1.08 | <0.001 |  |
| Female | 1.04 | 0.91 | 1.18 | 0.608 |  |
| Level of education | 0.99 | 0.98 | 1.00 | 0.087 |  |
| HT | 1.81 | 1.58 | 2.08 | <0.001 |  |
| DM | 1.10 | 0.96 | 1.26 | 0.163 |  |
| HLD | 1.19 | 1.04 | 1.36 | 0.012 |  |
| Hong Kong (reference) | / | / | / | <0.001 |  |
| Beijing | 0.95 | 0.60 | 1.51 | 0.816 |  |
| Bangkok | 2.08 | 1.38 | 3.14 | 0.001 |  |
| Bandung | 45.41 | 25.72 | 80.19 | <0.001 |  |
| Seoul Korea | 0.48 | 0.34 | 0.68 | <0.001 |  |
| Manilla | 0.26 | 0.15 | 0.44 | <0.001 |  |
| Singapore | 1.81 | 1.49 | 2.20 | <0.001 |  |
| Kaohsiung | 0.48 | 0.35 | 0.66 | <0.001 |  |
| Control (reference) | / | / | / | <0.001 |  |
| AD/MCI | 1.54 | 1.14 | 2.08 | 0.005 |  |
| Stroke/TIA | 1.90 | 1.49 | 2.43 | <0.001 |  |
| CT (reference) | / | / | / | <0.001 |  |
| MRI 1.5T | 1.37 | 1.00 | 1.86 | 0.047 |  |
| MRI 3T | 1.75 | 1.33 | 2.32 | <0.001 |  |
| **All subjects and Age <=60 (n=629)** | | | | |  |
| Dependent variables | OR | 95% CI | | **p* |  |
|  |  | Lower | Upper |  |  |
| Age | 1.05 | 1.01 | 1.10 | 0.017 |  |
| Female | 1.06 | 0.66 | 1.69 | 0.809 |  |
| Level of education | 0.99 | 0.94 | 1.04 | 0.725 |  |
| HT | 2.33 | 1.42 | 3.83 | 0.001 |  |
| DM | 1.29 | 0.79 | 2.12 | 0.307 |  |
| HLD | 1.11 | 0.69 | 1.79 | 0.676 |  |
| Hong Kong (reference) | / | / | / | <0.001 |  |
| Beijing | 0.34 | 0.03 | 3.90 | 0.385 |  |
| Bangkok | 2.39 | 1.08 | 5.29 | 0.031 |  |
| Bandung | 30.89 | 13.41 | 71.17 | <0.001 |  |
| Seoul Korea | 0.28 | 0.03 | 2.42 | 0.248 |  |
| Manilla | 0.11 | 0.01 | 1.78 | 0.119 |  |
| Singapore | 1.33 | 0.23 | 7.69 | 0.748 |  |
| Kaohsiung | 0.83 | 0.36 | 1.90 | 0.662 |  |
| Control (reference) | / | / | / | 0.038 |  |
| AD/MCI | 0.37 | 0.06 | 2.34 | 0.294 |  |
| Stroke/TIA | 0.21 | 0.06 | 0.71 | 0.012 |  |
| CT (reference) | / | / | / | 0.688 |  |
| MRI 1.5T | 0.73 | 0.31 | 1.72 | 0.474 |  |
| MRI 3T | 1.06 | 0.51 | 2.21 | 0.887 |  |
| **All subjects and Age 61-70 (n=1739)** | | | | |  |
| Dependent variables | OR | 95% CI | | **p* |  |
|  |  | Lower | Upper |  |  |
| Age | 1.05 | 1.00 | 1.09 | 0.041 |  |
| Female | 1.10 | 0.87 | 1.39 | 0.438 |  |
| Level of education | 0.97 | 0.95 | 1.00 | 0.028 |  |
| HT | 2.24 | 1.72 | 2.92 | <0.001 |  |
| DM | 1.25 | 0.98 | 1.59 | 0.076 |  |
| HLD | 1.28 | 1.00 | 1.64 | 0.049 |  |
| Hong Kong (reference) | / | / | / | <0.001 |  |
| Beijing | 0.57 | 0.22 | 1.46 | 0.242 |  |
| Bangkok | 3.22 | 1.63 | 6.37 | 0.001 |  |
| Bandung | 146.64 | 19.00 | 1131.75 | <0.001 |  |
| Seoul Korea | 0.28 | 0.15 | 0.55 | <0.001 |  |
| Manilla | 0.13 | 0.03 | 0.49 | 0.003 |  |
| Singapore | 1.27 | 0.94 | 1.71 | 0.121 |  |
| Kaohsiung | 0.48 | 0.27 | 0.86 | 0.013 |  |
| Control (reference) | / | / | / | 0.001 |  |
| AD/MCI | 0.78 | 0.40 | 1.49 | 0.446 |  |
| Stroke/TIA | 0.45 | 0.26 | 0.77 | 0.004 |  |
| CT (reference) | / | / | / | <0.001 |  |
| MRI 1.5T | 2.16 | 1.15 | 4.06 | 0.017 |  |
| MRI 3T | 3.04 | 1.77 | 5.22 | <0.001 |  |
| **All subjects and Age 71-80 (n=1980)** | | | | |  |
| Dependent variables | OR | 95% CI | | **p* |  |
|  |  | Lower | Upper |  |  |
| Age | 1.07 | 1.04 | 1.11 | <0.001 |  |
| Female | 0.93 | 0.76 | 1.14 | 0.471 |  |
| Level of education | 0.99 | 0.97 | 1.01 | 0.537 |  |
| HT | 1.59 | 1.28 | 1.97 | <0.001 |  |
| DM | 1.04 | 0.85 | 1.28 | 0.691 |  |
| HLD | 1.13 | 0.92 | 1.40 | 0.245 |  |
| Hong Kong (reference) | / | / | / | <0.001 |  |
| Beijing | 1.55 | 0.82 | 2.94 | 0.178 |  |
| Bangkok | 1.08 | 0.44 | 2.70 | 0.863 |  |
| Bandung | 38.07 | 5.01 | 289.30 | <0.001 |  |
| Seoul Korea | 1.02 | 0.62 | 1.67 | 0.945 |  |
| Manilla | 0.63 | 0.30 | 1.32 | 0.218 |  |
| Singapore | 2.67 | 1.95 | 3.65 | <0.001 |  |
| Kaohsiung | 0.44 | 0.25 | 0.76 | 0.003 |  |
| Control (reference) | / | / | / | 0.003 |  |
| AD/MCI | 2.38 | 1.31 | 4.31 | 0.004 |  |
| Stroke/TIA | 1.13 | 0.73 | 1.74 | 0.596 |  |
| CT (reference) | / | / | / | 0.060 |  |
| MRI 1.5T | 1.42 | 0.88 | 2.31 | 0.151 |  |
| MRI 3T | 1.79 | 1.10 | 2.91 | 0.019 |  |
| **All subjects and Age >=81 (n=867)** | | | | |  |
| Dependent variables | OR | 95% CI | | **p* |  |
|  |  | Lower | Upper |  |  |
| Age | 1.06 | 1.01 | 1.10 | 0.012 |  |
| Female | 1.04 | 0.75 | 1.44 | 0.807 |  |
| Level of education | 1.02 | 0.99 | 1.05 | 0.228 |  |
| HT | 1.49 | 1.08 | 2.04 | 0.014 |  |
| DM | 0.86 | 0.62 | 1.18 | 0.343 |  |
| HLD | 1.16 | 0.83 | 1.62 | 0.382 |  |
| Hong Kong (reference) | / | / | / | <0.001 |  |
| Beijing | 1.44 | 0.30 | 6.88 | 0.646 |  |
| Bangkok | 0.56 | 0.16 | 1.91 | 0.352 |  |
| Bandung | 1.88 | 0.18 | 19.24 | 0.594 |  |
| Seoul Korea | 0.47 | 0.14 | 1.61 | 0.23 |  |
| Manilla | 0.20 | 0.05 | 0.78 | 0.021 |  |
| Singapore | 2.54 | 1.13 | 5.68 | 0.024 |  |
| Kaohsiung | 0.19 | 0.08 | 0.44 | <0.001 |  |
| Control (reference) | / | / | / | 0.023 |  |
| AD/MCI | 2.86 | 0.73 | 11.23 | 0.133 |  |
| Stroke/TIA | 0.87 | 0.29 | 2.59 | 0.801 |  |
| CT (reference) | / | / | / | 0.105 |  |
| MRI 1.5T | 1.75 | 0.77 | 4.02 | 0.184 |  |
| MRI 3T | 2.26 | 1.06 | 4.81 | 0.034 |  |
| Supplementary Table S4 showed the risk factors of moderate-to-severe WML in different age groups (all subjects). Age, sex, level of education, Asian cities, disease group, and imaging modality were entered as covariates in each regression model. Abbreviations: DM = diabetes mellitus; HLD = hyperlipidemia; HT = hypertension. | | | | |  |
|  |  |  |  |  |  |
| ***** Bonferroni correction is applied to the alpha (*α* = 0.01). | | | |  |  |

**Supplementary Table S5**

| **Stroke/TIA (n=1786)** | | | | |  |
| --- | --- | --- | --- | --- | --- |
| Dependent variables | OR | 95% CI | | **p* |  |
|  |  | Lower | Upper |  |  |
| Age | 1.07 | 1.06 | 1.08 | <0.001 |  |
| Female | 1.26 | 1.01 | 1.57 | 0.040 |  |
| Level of education | 0.99 | 0.97 | 1.02 | 0.493 |  |
| HT | 1.79 | 1.40 | 2.30 | <0.001 |  |
| DM | 1.08 | 0.87 | 1.35 | 0.482 |  |
| HLD | 1.21 | 0.97 | 1.51 | 0.092 |  |
| Hong Kong (reference) | / | / | / | 0.000 |  |
| Bangkok | 1.76 | 1.15 | 2.70 | 0.010 |  |
| Bandung | 44.01 | 24.28 | 79.77 | <0.001 |  |
| Singapore | 0.72 | 0.44 | 1.19 | 0.200 |  |
| Kaohsiung | 0.37 | 0.26 | 0.52 | <0.001 |  |
| CT (reference) | / | / | / | <0.001 |  |
| MRI 1.5T | 1.23 | 0.89 | 1.70 | 0.216 |  |
| MRI 3T | 2.19 | 1.59 | 3.00 | <0.001 |  |
| **Stroke/TIA and Age <=60 (n=484)** | | | | |  |
| Dependent variables | OR | 95% CI | | **p* |  |
|  |  | Lower | Upper |  |  |
| Age | 1.06 | 1.02 | 1.11 | 0.008 |  |
| Female | 0.89 | 0.53 | 1.48 | 0.646 |  |
| Level of education | 1.00 | 0.94 | 1.05 | 0.886 |  |
| HT | 2.34 | 1.35 | 4.08 | 0.003 |  |
| DM | 1.02 | 0.60 | 1.75 | 0.937 |  |
| HLD | 1.03 | 0.61 | 1.73 | 0.911 |  |
| Hong Kong (reference) | / | / | / | <0.001 |  |
| Bangkok | 2.36 | 1.07 | 5.23 | 0.035 |  |
| Bandung | 29.08 | 12.62 | 67.01 | <0.001 |  |
| Singapore | 1.29 | 0.23 | 7.41 | 0.775 |  |
| Kaohsiung | 0.85 | 0.37 | 1.94 | 0.697 |  |
| CT (reference) | / | / | / | 0.548 |  |
| MRI 1.5T | 0.67 | 0.28 | 1.61 | 0.368 |  |
| MRI 3T | 1.09 | 0.52 | 2.27 | 0.824 |  |
| **Stroke/TIA and Age 61-70 (n=520)** | | | | |  |
| Dependent variables | OR | 95% CI | | **p* |  |
|  |  | Lower | Upper |  |  |
| Age | 0.99 | 0.92 | 1.07 | 0.861 |  |
| Female | 1.43 | 0.92 | 2.23 | 0.110 |  |
| Level of education | 0.99 | 0.95 | 1.03 | 0.625 |  |
| HT | 1.93 | 1.19 | 3.13 | 0.007 |  |
| DM | 1.13 | 0.74 | 1.73 | 0.563 |  |
| HLD | 1.40 | 0.91 | 2.15 | 0.130 |  |
| Hong Kong (reference) | / | / | / | <0.001 |  |
| Bangkok | 2.51 | 1.22 | 5.14 | 0.012 |  |
| Bandung | 153.07 | 19.62 | 1194.45 | <0.001 |  |
| Singapore | 0.42 | 0.19 | 0.92 | 0.030 |  |
| Kaohsiung | 0.34 | 0.18 | 0.65 | 0.001 |  |
| CT (reference) | / | / | / | <0.001 |  |
| MRI 1.5T | 1.86 | 0.98 | 3.52 | 0.056 |  |
| MRI 3T | 4.22 | 2.34 | 7.61 | <0.001 |  |
| **Stroke/TIA and Age 71-80 (n=518)** | | | | |  |
| Dependent variables | OR | 95% CI | | **p* |  |
|  |  | Lower | Upper |  |  |
| Age | 1.06 | 0.99 | 1.13 | 0.098 |  |
| Female | 1.33 | 0.91 | 1.96 | 0.140 |  |
| Level of education | 0.99 | 0.95 | 1.03 | 0.669 |  |
| HT | 1.65 | 1.05 | 2.59 | 0.029 |  |
| DM | 1.11 | 0.76 | 1.63 | 0.584 |  |
| HLD | 1.16 | 0.79 | 1.69 | 0.450 |  |
| Hong Kong (reference) | / | / | / | <0.001 |  |
| Bangkok | 0.90 | 0.35 | 2.30 | 0.823 |  |
| Bandung | 35.83 | 4.68 | 274.28 | 0.001 |  |
| Singapore | 1.14 | 0.47 | 2.78 | 0.775 |  |
| Kaohsiung | 0.34 | 0.18 | 0.66 | 0.001 |  |
| CT (reference) | / | / | / | 0.059 |  |
| MRI 1.5T | 1.29 | 0.76 | 2.16 | 0.344 |  |
| MRI 3T | 2.03 | 1.13 | 3.65 | 0.018 |  |
| **Stroke/TIA and Age >=81 (n=264)** | | | | |  |
| Dependent variables | OR | 95% CI | | **p* |  |
|  |  | Lower | Upper |  |  |
| Age | 1.11 | 1.03 | 1.20 | 0.007 |  |
| Female | 1.27 | 0.73 | 2.23 | 0.400 |  |
| Level of education | 1.03 | 0.97 | 1.10 | 0.334 |  |
| HT | 1.23 | 0.65 | 2.33 | 0.529 |  |
| DM | 1.03 | 0.59 | 1.81 | 0.918 |  |
| HLD | 1.14 | 0.67 | 1.96 | 0.628 |  |
| Hong Kong (reference) | / | / | / | 0.010 |  |
| Bangkok | 0.52 | 0.15 | 1.86 | 0.316 |  |
| Bandung | 2.01 | 0.19 | 20.85 | 0.557 |  |
| Singapore | / | / | / | 0.999 |  |
| Kaohsiung | 0.17 | 0.07 | 0.45 | <0.001 |  |
| CT (reference) | / | / | / | 0.087 |  |
| MRI 1.5T | 1.83 | 0.66 | 5.12 | 0.246 |  |
| MRI 3T | 2.52 | 1.04 | 6.07 | 0.040 |  |
| Supplementary Table S5 showed the risk factors of moderate-to-severe WML in different age groups (Stroke/TIA). Age, sex, level of education, Asian cities, and imaging modality were entered as covariates in each regression model. | | | | |  |
|  |  |  |  |  |  |
| Abbreviations: DM = diabetes mellitus; HLD = hyperlipidemia; HT = hypertension. | | | | |  |
| ***** Bonferroni correction is applied to the alpha (*α* = 0.01). | | | |  |  |

**Supplementary Table S6**

| **AD/MCI (n=1633)** | | | | |  |
| --- | --- | --- | --- | --- | --- |
| Dependent variables | OR | 95% CI | | **p* |  |
|  |  | Lower | Upper |  |  |
| Age | 1.06 | 1.05 | 1.08 | <0.001 |  |
| Female | 0.81 | 0.63 | 1.04 | 0.095 |  |
| Level of education | 1.01 | 0.98 | 1.03 | 0.643 |  |
| HT | 1.76 | 1.40 | 2.22 | <0.001 |  |
| DM | 1.07 | 0.83 | 1.38 | 0.602 |  |
| HLD | 1.12 | 0.86 | 1.45 | 0.404 |  |
| Beijing (reference) | / | / | / | <0.001 |  |
| Seoul Korea | 0.55 | 0.38 | 0.81 | 0.002 |  |
| Manilla | 0.29 | 0.17 | 0.48 | <0.001 |  |
| Singapore | 2.71 | 1.69 | 4.35 | <0.001 |  |
| CT (reference) | / | / | / | 0.993 |  |
| MRI 1.5T | / | / | / | 0.999 |  |
| MRI 3T | / | / | / | 0.999 |  |
| **AD/MCI and Age <=60 (n=85)** | | | | |  |
| Dependent variables | OR | 95% CI | | **p* |  |
|  |  | Lower | Upper |  |  |
| Age | 0.98 | 0.80 | 1.20 | 0.837 |  |
| Female | 2.37 | 0.57 | 9.95 | 0.238 |  |
| Level of education | 0.88 | 0.71 | 1.10 | 0.268 |  |
| HT | 3.78 | 0.82 | 17.55 | 0.089 |  |
| DM | 4.71 | 0.92 | 24.02 | 0.062 |  |
| HLD | 0.77 | 0.13 | 4.57 | 0.774 |  |
| Beijing (reference) | / | / | / | 0.126 |  |
| Seoul Korea | 0.47 | 0.04 | 5.19 | 0.539 |  |
| Manilla | 0.22 | 0.01 | 4.27 | 0.315 |  |
| Singapore | 2.93 | 0.45 | 18.91 | 0.258 |  |
| MRI 1.5T (reference) | / | / | / |  |  |
| MRI 3T | 0.30 | 0.01 | 6.81 | 0.446 |  |
| **AD/MCI and Age 61-70 (n=285)** | | | | |  |
| Dependent variables | OR | 95% CI | | **p* |  |
|  |  | Lower | Upper |  |  |
| Age | 1.14 | 1.00 | 1.30 | 0.045 |  |
| Female | 0.92 | 0.46 | 1.83 | 0.806 |  |
| Level of education | 0.98 | 0.91 | 1.06 | 0.620 |  |
| HT | 3.25 | 1.60 | 6.61 | 0.001 |  |
| DM | 0.73 | 0.33 | 1.62 | 0.433 |  |
| HLD | 0.78 | 0.38 | 1.61 | 0.508 |  |
| Beijing (reference) | / | / | / | <0.001 |  |
| Seoul Korea | 0.36 | 0.14 | 0.95 | 0.040 |  |
| Manilla | 0.22 | 0.05 | 0.95 | 0.043 |  |
| Singapore | 3.86 | 1.40 | 10.67 | 0.009 |  |
| CT (reference) | / | / | / | 0.385 |  |
| MRI 1.5T | / | / | / | 0.999 |  |
| MRI 3T | / | / | / | 0.999 |  |
| **AD/MCI and Age 71-80 (n=772)** | | | | |  |
| Dependent variables | OR | 95% CI | | **p* |  |
|  |  | Lower | Upper |  |  |
| Age | 1.09 | 1.03 | 1.16 | 0.003 |  |
| Female | 0.66 | 0.46 | 0.94 | 0.021 |  |
| Level of education | 1.00 | 0.97 | 1.04 | 0.919 |  |
| HT | 1.52 | 1.09 | 2.11 | 0.013 |  |
| DM | 1.12 | 0.79 | 1.59 | 0.531 |  |
| HLD | 1.17 | 0.82 | 1.68 | 0.389 |  |
| Beijing (reference) | / | / | / | <0.001 |  |
| Seoul Korea | 0.66 | 0.40 | 1.08 | 0.097 |  |
| Manilla | 0.40 | 0.20 | 0.83 | 0.013 |  |
| Singapore | 2.56 | 1.32 | 4.98 | 0.005 |  |
| CT (reference) | / | / | / | 0.819 |  |
| MRI 1.5T | / | / | / | 0.999 |  |
| MRI 3T | / | / | / | 0.999 |  |
| **AD/MCI and Age >=81 (n=491)** | | | | |  |
| Dependent variables | OR | 95% CI | | **p* |  |
|  |  | Lower | Upper |  |  |
| Age | 1.04 | 0.98 | 1.10 | 0.201 |  |
| Female | 0.89 | 0.56 | 1.42 | 0.627 |  |
| Level of education | 1.02 | 0.98 | 1.07 | 0.264 |  |
| HT | 1.57 | 1.05 | 2.34 | 0.027 |  |
| DM | 0.89 | 0.56 | 1.39 | 0.600 |  |
| HLD | 1.18 | 0.72 | 1.93 | 0.504 |  |
| Beijing (reference) | / | / | / | <0.001 |  |
| Seoul Korea | 0.33 | 0.12 | 0.94 | 0.037 |  |
| Manilla | 0.13 | 0.04 | 0.44 | 0.001 |  |
| Singapore | 1.72 | 0.43 | 6.87 | 0.440 |  |
| MRI 1.5T (reference) | / | / | / |  |  |
| MRI 3T | 1.23 | 0.52 | 2.94 | 0.637 |  |
| Supplementary Table S6 showed the risk factors of moderate-to-severe WML in different age groups (AD/MCI). Age, sex, level of education, Asian cities, and imaging modality were entered as covariates in each regression model. | | | | |  |
|  |  |  |  |  |  |
| Abbreviations: DM = diabetes mellitus; HLD = hyperlipidemia; HT = hypertension. | | | | |  |
|  |  |  |  |  |  |
| ***** Bonferroni correction is applied to the alpha (*α* = 0.01). | | | |  |  |

**Supplementary Table S7**

| **Controls (n=1796)** | | | | |  |
| --- | --- | --- | --- | --- | --- |
| Dependent variables | OR | 95% CI | | **p* |  |
|  |  | Lower | Upper |  |  |
| Age | 1.10 | 1.08 | 1.12 | <0.001 |  |
| Female | 0.94 | 0.75 | 1.18 | 0.591 |  |
| Level of education | 0.98 | 0.96 | 1.01 | 0.184 |  |
| HT | 1.97 | 1.52 | 2.55 | <0.001 |  |
| DM | 1.15 | 0.91 | 1.45 | 0.233 |  |
| HLD | 1.12 | 0.88 | 1.43 | 0.356 |  |
| Hong Kong (reference) | / | / | / | <0.001 |  |
| Seoul Korea | 1.13 | 0.65 | 1.95 | 0.671 |  |
| Singapore | 2.18 | 1.71 | 2.79 | <0.001 |  |
| **Controls and Age <=60 (n=60)** | | | | |  |
| Dependent variables | OR | 95% CI | | **p* |  |
|  |  | Lower | Upper |  |  |
| Female | 7.87 | 0.43 | 144.91 | 0.165 |  |
| Level of education | 1.08 | 0.76 | 1.52 | 0.672 |  |
| HT | 0.12 | 0.01 | 2.05 | 0.141 |  |
| DM | 8.40 | 0.39 | 182.94 | 0.176 |  |
| HLD | 7.00 | 0.35 | 138.69 | 0.202 |  |
| Singapore | 12.04 | 0.54 | 269.50 | 0.117 |  |
| **Controls and Age 61-70 (n=934)** | | | | |  |
| Dependent variables | OR | 95% CI | | **p* |  |
|  |  | Lower | Upper |  |  |
| Age | 1.08 | 1.02 | 1.15 | 0.016 |  |
| Female | 0.87 | 0.63 | 1.21 | 0.416 |  |
| Level of education | 0.97 | 0.94 | 1.01 | 0.109 |  |
| HT | 2.30 | 1.59 | 3.34 | 0.000 |  |
| DM | 1.54 | 1.10 | 2.15 | 0.012 |  |
| HLD | 1.17 | 0.82 | 1.66 | 0.383 |  |
| Hong Kong (reference) | / | / | / | 0.016 |  |
| Seoul Korea | 0.70 | 0.28 | 1.74 | 0.440 |  |
| Singapore | 1.63 | 1.12 | 2.37 | 0.010 |  |
| **Controls and Age 61-80 (n=690)** | | | | |  |
| Dependent variables | OR | 95% CI | | **p* |  |
|  |  | Lower | Upper |  |  |
| Age | 1.08 | 1.01 | 1.15 | 0.015 |  |
| Female | 0.95 | 0.67 | 1.34 | 0.765 |  |
| Level of education | 1.00 | 0.96 | 1.03 | 0.781 |  |
| HT | 1.79 | 1.20 | 2.67 | 0.005 |  |
| DM | 0.92 | 0.65 | 1.31 | 0.644 |  |
| HLD | 1.07 | 0.74 | 1.55 | 0.725 |  |
| Hong Kong (reference) | / | / | / | <0.001 |  |
| Seoul Korea | 2.63 | 1.11 | 6.21 | 0.028 |  |
| Singapore | 2.88 | 1.99 | 4.16 | 0.000 |  |
| **Controls and Age >=81 (n=112)** | | | | |  |
| Dependent variables | OR | 95% CI | | **p* |  |
|  |  | Lower | Upper |  |  |
| Age | 0.98 | 0.79 | 1.22 | 0.858 |  |
| Female | 1.29 | 0.51 | 3.24 | 0.590 |  |
| Level of education | 0.99 | 0.89 | 1.10 | 0.817 |  |
| HT | 1.71 | 0.59 | 4.96 | 0.321 |  |
| DM | 0.52 | 0.21 | 1.29 | 0.158 |  |
| HLD | 1.33 | 0.49 | 3.61 | 0.576 |  |
| Singapore | 2.22 | 0.85 | 5.77 | 0.102 |  |
| Supplementary Table S7 showed the risk factors of moderate-to-severe WML in different age groups (controls). Age, sex, level of education and Asian cities were entered as covariates in each regression model. | | | | |  |
|  |  |  |  |  |  |
| Abbreviations: DM = diabetes mellitus; HLD = hyperlipidemia; HT = hypertension. | | | | |  |
|  |  |  |  |  |  |
| ***** Bonferroni correction is applied to the alpha (*α* = 0.01). | | | |  |  |

**References**

1 Cicchetti, D. V. Guidelines, criteria, and rules of thumb for evaluating normed and standardized assessment instruments in psychology. *Psychological assessment* **6**, 284 (1994).
